# Unmasking a Hidden Bridge: Exercise‐Induced Right Bundle Branch Block Without Ischemia

**DOI:** 10.1155/cric/2330053

**Published:** 2026-08-07

**Authors:** Ana Karen Nieto-Dolores, Hugo Armando Valencia-Hernandez, Alfonso Gonzalez-Trejo, Nilda Espinola-Zavaleta, Enrique C. Guerra, Maria Jose Santa-Ana-Bayona, Pavel Martinez-Dominguez, Gilberto H. Gutierrez-Acosta, Camila Ponce-Acosta

**Affiliations:** ^1^ Faculty of Medicine, Popular Autonomous University of Puebla, Puebla City, Mexico; ^2^ School of Medicine, Villa Rica University, Veracruz, Mexico; ^3^ Faculty of Medicine, National Autonomous University of Mexico, Mexico City, Mexico, unam.mx; ^4^ Department of Nuclear Cardiology, National Institute of Cardiology Ignacio Chavez, Mexico City, Mexico; ^5^ MD-PhD (PECEM) Program, Faculty of Medicine, National Autonomous University of Mexico, Mexico City, Mexico, unam.mx; ^6^ Mexican Faculty of Medicine, La Salle University, Mexico City, Mexico, lasalle.edu; ^7^ Department of Cardiovascular Medicine, Mayo Clinic, Rochester, Minnesota, USA, mayo.edu; ^8^ Institute of Biomedical Science, Autonomous University of Juarez City, Nuevo Casas Grandes, Chihuahua, Mexico, uacj.mx

**Keywords:** coronary CT angiography, exercise stress test, myocardial bridging, rate-dependent conduction abnormality, right bundle branch block

## Abstract

**Background:**

Myocardial bridging (MB) is usually benign but may occasionally be associated with ischemia or arrhythmias. Exercise induced conduction abnormalities are rare and poorly understood.

**Case Summary:**

A 69‐year‐old asymptomatic male developed transient right bundle branch block (RBBB) at peak exertion during treadmill stress testing, without angina, ischemic ECG changes, or hemodynamic instability. The RBBB resolved immediately during recovery. Coronary computed tomography angiography identified a superficial myocardial bridge of the mid‐left anterior descending artery without luminal narrowing or atherosclerosis.

**Discussion:**

The findings supported a benign, rate dependent conduction delay rather than ischemia.

**Conclusion:**

This case illustrates a rare coexistence of MB with exercise‐induced RBBB and highlights the importance of recognizing nonischemic conduction phenomena to avoid unnecessary invasive studies.

**Take Home Messages**

• Exercise‐induced right bundle branch block (EI‐RBBB) is an exceptionally rare finding during stress testing and is often rate‐dependent rather than ischemic in origin.
• Myocardial bridging (MB) of the left anterior descending (LAD) artery can coexist with conduction disturbances without producing hemodynamic compromise or myocardial ischemia.
• CT angiography helps exclude atherosclerosis and avoids unnecessary invasive angiography.
• Recognizing nonischemic conduction phenomena reduces patient risk and healthcare cost.

## 1. Introduction

MB refers to an anatomical variant in which a segment of a coronary artery tunnels through the myocardium rather than resting on its surface [1]. Although most bridges are asymptomatic, systolic compression can occasionally lead to ischemia, arrhythmias, or myocardial infarction. Reported prevalence varies from 3%–7% on angiography and up to 25% in autopsy studies [2]. Exercise‐induced conduction abnormalities are rare, and EI‐RBBB is significantly less frequent than exercise‐induced left bundle branch block (EI‐LBBB). The coexistence of EI‐RBBB with MB has not been previously well described.

## 2. History of Presentation

**Figure 1:**
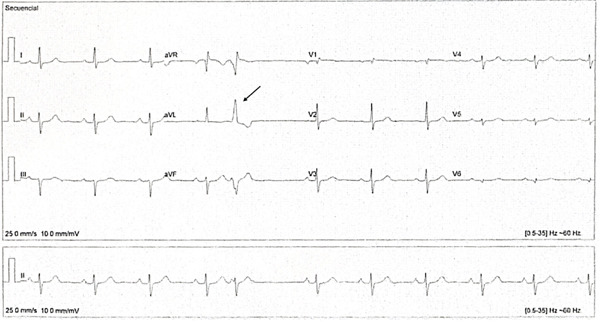
Baseline electrocardiogram: Baseline electrocardiogram showing ventricular extrasystole (arrow).

**Figure 2:**
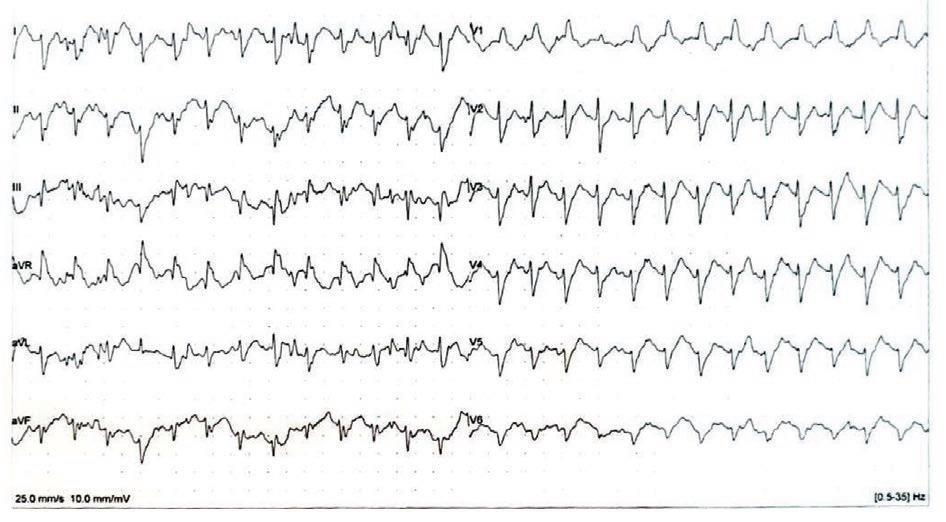
Exercise stress test electrocardiogram: Twelve lead electrocardiogram obtained during the maximal exertion, demonstrating an incomplete right bundle branch block.

A 69‐year‐old male patient attended his routine monthly follow‐up. He was asymptomatic and denied any chest pain, palpitations, or dyspnea. A baseline electrocardiogram (ECG) **(**Figure 1 **)** showed ventricular extrasystoles in V1–V2, prompting an exercise stress test. During exercise, a new onset right bundle branch block (RBBB) appeared at a heart rate of 132 bpm, resolving promptly during early recovery at 118 bpm **(**Figure 2 **)**. No chest pain, ischemic changes, arrhythmias, or hemodynamic abnormalities occurred.

### 2.1. Past Medical History

Prior medical history included hypertension diagnosed 7 years ago, currently managed with bisoprolol 2.5 mg daily and telmisartan 80 mg daily, and hypercholesterolemia and hypertriglyceridemia receiving treatment with ezetimibe and atorvastatin once daily.

### 2.2. Differential Diagnosis

The appearance of an exercise‐induced RBBB raised suspicion for possible ischemic heart disease, since exertional conduction changes may signal underlying coronary pathology. However, the absence of angina, normal perfusion imaging, and negative ischemic response argued against coronary artery disease. Other potential causes such as myocarditis, electrolyte disturbances, and drug‐induced delay were excluded based on the absence of systemic symptoms, normal laboratory studies, and no history of medication use affecting cardiac conduction. Structural abnormalities, including hypertrophic cardiomyopathy or infiltrative diseases such as sarcoidosis or amyloidosis, were also ruled out by echocardiography.

### 2.3. Investigations

**Figure 3:**
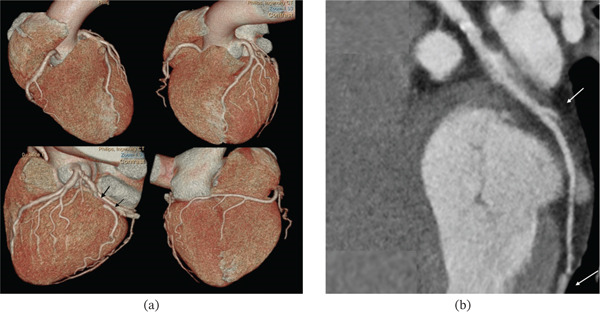
(a) Three‐dimensional volume reconstruction of the heart and coronary arteries. Multiple three‐dimensional volume rendered images obtained from coronary computed tomography angiography illustrating the intramyocardial course of the mid‐segment of the LAD. The vessel is traversing beneath a thin layer of myocardial fibers before returning to the epicardial surface distally. (b) 2D‐curved multiplanar reformation images. In the mid‐segment of the LAD coursing intramyocardially, a myocardial bridging is observed (arrows). No evidence of luminal narrowing or atherosclerotic involvement is seen in the bridged or adjacent segments of the LAD.

Coronary CT angiography (Figure 3a,b) revealed a superficial mid‐LAD myocardial bridge with preserved lumen and no atherosclerosis. No structural abnormalities were seen.

### 2.4. Management (Medical/Interventions)

Given the patient remained completely asymptomatic and no evidence of myocardial ischemia or structural heart disease was identified, no specific therapeutic intervention was required. A conservative approach with observation and regular follow‐up was adopted. The patient was advised to continue routine cardiovascular risk factor control and periodic cardiology evaluations. Pharmacological management included atorvastatin 40 mg daily and aspirin 100 mg daily as part of cardiovascular prevention. In the absence of symptoms or ischemic findings, further medical or surgical treatment was not indicated.

### 2.5. Outcome and Follow‐Up

The patient remained asymptomatic during follow‐up, without recurrent arrhythmias or symptoms. Conservative management with periodic cardiology evaluation was continued.

## 3. Discussion

MB is a congenital anatomical variant in which a coronary artery segment courses intramyocardially, rather than on the epicardial surface. The LAD is affected in up to 67%–98% of cases. Three morphological patterns are described depending on depth and fiber orientation: superficial myocardial fibers traversing over the LAD, deeper fibers partially or completely encircling the artery, and bridges deeper than 5 mm, which are less amenable to surgical myotomy [3]. In this case, coronary CT angiography revealed a mild, superficial bridge of the LAD without atherosclerotic or hemodynamic obstruction.

The physiological impact of MB varies with the cardiac cycle, heart rate and sympathetic tone [4]. During systole, myocardial fibers compress the tunneled segment causing a transient, phasic narrowing of the vessel, whereas in diastole, the lumen partially re‐expands. This cyclical compression may delay early diastolic hyperemia and limit subendocardial perfusion, particularly during tachycardia or increased myocardial demand, predisposing to ischemia [4]. However, our patient remained entirely asymptomatic during the exercise test, suggesting that the bridge was hemodynamically insignificant under stress.

Angiographically, MB is identified by a change in vessel diameter between systole and diastole within the bridged segment, known as the “milking effect” characterized by a ≥ 70% reduction in luminal diameter during systole with persistent narrowing ≥ 35% in diastole [4].

Complementary modalities such as intravascular ultrasound may reveal a “half moon” echolucent space between the bridged segment and the epicardium confirming the intramyocardial course [3]. In this case, coronary CT angiography confirmed a bridged LAD segment without significant luminal narrowing or ischemia, consistent with a benign anatomical variant.

Most myocardial bridges are asymptomatic and discovered incidentally. Nevertheless, MB has been associated with exertional angina, arrhythmias, and even myocardial infarction when compression impairs diastolic coronary filling [1]. In our patient, the unique finding was the development of an EI‐RBBB during treadmill testing without accompanying symptoms or evidence of ischemia.

Conduction disturbances provoked by exercise testing are uncommon. EI‐LBBB is more frequently reported, with an estimated prevalence of 0.38% in large cohorts [5]. In contrast, EI‐RBBB is even rarer, occurring in approximately 0.24% of patients and not independently associated with increased mortality after adjustment for age and comorbidities [5].

A directed literature search identified very few published cases of isolated EI‐RBBB, and importantly, none describing its coexistence with MB as the only structural abnormality. This increases the novelty and educational value of the present case.

The mechanism of EI‐RBBB is generally attributed to rate‐dependent intraventricular conduction delay, where rapid heart rate shortens refractory periods heterogeneously, leading to functional block in fibers of the right bundle.

In our patient the RBBB appeared at a heart rate of approximately 132 bpm and resolved promptly during early recovery at 118 bpm, strongly supporting a tachycardia dependent or Phase 3 block mechanism, which is well described in conduction physiology [6]. The absence of symptoms, ischemia, arrhythmias, or hemodynamic abnormalities further supports this benign interpretation. Some authors propose that rate‐dependent RBBB may reflect underlying subclinical conduction system disease; although in most cases (including the present one) patients remain asymptomatic with a favorable prognosis.

Although MB does not directly involve the conduction system, a theoretical relationship exists. The LAD gives rise to septal perforator arteries that supply the right bundle branch. During tachycardia, transient changes in septal perfusion or wall stress (especially in the setting of MB) may subtly modulate intramyocardial conduction without producent overt ischemia.

Although speculative, this physiologic hypothesis may explain why the conduction abnormality manifested only under increased myocardial demand.

Importantly, the presence of EI‐RBBB in this patient did not reflect structural heart disease or ischemia, as demonstrated by normal perfusion, normal hemodynamic response, and normal findings aside from a mild MB. Recognizing this benign pattern prevents unnecessary invasive evaluation.

Management of MB depends on the clinical presentation and hemodynamic relevance of the bridge. In asymptomatic patients like ours, a conservative approach based on observation and control of cardiovascular risk factors is generally sufficient. For symptomatic patients, B‐blockers remain the main conservative treatment; calcium channel blockers may have vasodilatory effects beneficial for the concomitant vasospasm [7]. Nitrates are contraindicated in patients with MB. In the present case, given the absence of ischemia and the benign course, a noninvasive strategy with close follow‐up was appropriate. The patient was maintained on atorvastatin 40 mg daily, ezetimibe 10 mg daily, ACE‐inhibitor and aspirin (aspirin protect) as part of cardiovascular risk reduction therapy and continued regular cardiology evaluations and aspirin.

## 4. Conclusion

This case highlights a rare coexistence of MB with exercise‐induced RBBB in an asymptomatic patient without ischemia. Recognition of rate‐dependent conduction delay is crucial to avoid unnecessary invasive testing. Coronary CT angiography plays an important role in excluding structural disease and guiding conservative management.

**Nomenclature**
CT: computed tomography
ECG: electrocardiogram
EI‐LBBB: exercise‐induced left bundle branch block
EI‐RBBB: exercise‐induced right bundle branch block
LAD: left anterior descending artery
MB: myocardial bridging
RBBB: right bundle branch block

## Funding

No funding was received for this manuscript.

## Disclosure

The authors have nothing to report.

## Consent

Written informed consent for publication was obtained.

## Conflicts of Interest

The authors declare no conflicts of interest.

## Data Availability

The data supporting this case report are available from the corresponding author upon reasonable request. Data availability is subject to necessary restrictions to protect patient privacy and confidentiality.

## References

[1] Evbayekha E. O. , Nwogwugwu E. , Olawoye A. , Bolaji K. , Adeosun A. A. , Ajibowo A. O. , Nsofor G. C. , Chukwuma V. N. , Shittu H. O. , Onuegbu C. A. , Adedoyin A. M. , and Okobi O. E. , A Comprehensive Review of Myocardial Bridging: Exploring Diagnostic and Treatment Modalities, Cureus. (2023) 15, no. 8, 10.7759/cureus.43132, 37692750. PMC10484041 37692750

[2] Shah S. M. , Quesada O. , and Henry T. D. , The Challenge of Myocardial Bridging, Journal of the Society for Cardiovascular Angiography & Interventions. (2023) 2, no. 2, 100545, 10.1016/j.jscai.2022.100545, 39129805. 39129805 PMC11308591

[3] Corban M. T. , Hung O. Y. , Eshtehardi P. , Rasoul-Arzrumly E. , McDaniel M. , Mekonnen G. , Timmins L. H. , Lutz J. , Guyton R. A. , and Samady H. , Myocardial Bridging, Journal of the American College of Cardiology. (2014) 63, no. 22, 2346–2355, 10.1016/j.jacc.2014.01.049, 24583304. 24583304 PMC4065198

[4] Sternheim D. , Power D. A. , Samtani R. , Kini A. , Fuster V. , and Sharma S. , Myocardial Bridging: Diagnosis, Functional Assessment, and Management, Journal of the American College of Cardiology. (2021) 78, no. 22, 2196–2212, 10.1016/j.jacc.2021.09.859. 34823663

[5] Stein R. , Ho M. , Oliveira C. M. , Ribeiro J. P. , Lata K. , and Abella J. , Heart, exercise and the Brazilian Archives of Cardiology, Arquivos Brasileiros de Cardiologia. (2011) 97, no. 6, 446–448, 22262146. 22262146 10.1590/s0066-782x2011001500002

[6] Rosenbaum M. B. , Elizari M. V. , and Lazzari J. O. , The Mechanism of Intermittent Bundle Branch Block: Phase 3 and PHASE 4 block, Progress in Cardiovascular Diseases. (1969) 11, no. 5, 438–466.

[7] Yuan S. M. , Myocardial Bridging, Brazilian Journal of Cardiovascular Surgery. (2016) 31, no. 1, 60–62, 10.5935/1678-9741.20150082. 27074276 PMC5062697

